# Recombinant Human SLPI Surface Functionalization Enhances Early Osseointegration and Biomechanical Stability of Titanium Implants in Rat Model

**DOI:** 10.3390/jfb17040205

**Published:** 2026-04-20

**Authors:** Wannapat Chouyratchakarn, Burin Boonsri, Surasak Tangkamonsri, Watchara Thepsupa, Chayarop Supanchart, Sarawut Kumphune

**Affiliations:** 1 Biomedical Engineering and Innovation Research Centre, CMU-BIOPOLIS Building, Chiang Mai University—Mae Hia Campus, Mae-Hia District, Chiang Mai 50100, Thailand; wannapat_ch@cmu.ac.th (W.C.); burin_boo@cmu.ac.th (B.B.); surasak_tangk@cmu.ac.th (S.T.); watcharath63@nu.ac.th (W.T.); chayarop.supanchart@cmu.ac.th (C.S.); 2 Biomedical Engineering Institute CMU-BIOPOLIS Building, Chiang Mai University—Mae Hia Campus, Mae-Hia District, Chiang Mai 50100, Thailand; 3 Department of Companion Animal and Wildlife Clinic, Faculty of Veterinary Medicine, Chiang Mai University—Mae Hia Campus, Mae-Hia District, Chiang Mai 50100, Thailand; 4 Department of Oral and Maxillofacial Surgery, Faculty of Dentistry, Chiang Mai University, Chiang Mai 50200, Thailand

**Keywords:** secretory leukocyte protease inhibitor (SLPI), titanium implant, osseointegration, surface modification

## Abstract

Titanium and its alloys are used in dental and orthopedic implants. However, long-term stability remains a clinical challenge. To overcome this limitation, surface modification has been investigated to improve surface properties. Our previous study demonstrated that the immobilization of secretory leukocyte protease inhibitor (SLPI) on the titanium surface promotes osteoblast adhesion, proliferation, and differentiation in vitro. The current study demonstrated the first in vivo evaluation of SLPI as a bioactive coating for medical implants. Grade 5 titanium screws were coated with 10 µg/mL of recombinant human SLPI (rhSLPI) for 24 h via simple physical adsorption, and the results were preliminarily validated via FE-SEM and ELISA. These SLPI-coated titanium screws (TiSs) were then placed in the tibia of Sprague–Dawley rats for 4 and 8 weeks. The hematological and biochemical parameters (BUN, Creatinine, AST, and Troponin I) demonstrated no acute systemic alterations within the 8-week period across all groups. Moreover, micro-computed tomography (micro-CT) and histological analysis revealed significantly higher bone volume fraction (%BV/TV) at 4 weeks compared to uncoated controls (20.64% ± 2.452% vs. 11.73% ± 0.524%). Finally, the biomechanical stability of implants, assessed using the removal torque test, showed that TiSs showed higher strength compared to Ti at both 4 and 8 weeks. In conclusion, this study represents a novel approach to transitioning rhSLPI-coated titanium evaluation from in vitro models to an in vivo rat model. rhSLPI surface functionalization enhances early-stage osseointegration and improves implant mechanical stability without acute hematological and biochemical alterations. These proof-of-concept findings suggest the potential of SLPI as a bioactive coating strategy.

## 1. Introduction

Titanium and its alloys are used as materials in dental and orthopedic implantation due to their surface properties, biocompatibility, and high short-term clinical success rate [1]. However, long-term stability remains a clinical challenge [2]. The long-term success of Ti implants relies on rapid osseointegration, which refers to the structural attachment and quantity of bone mass integrated around the implant surface [3]. To overcome limitations, surface modification has been studied for decades [4]. To enhance osseointegration, surface modification has been developed to improve physicochemical surface properties such as surface topology and surface hydrophilicity [1]. Furthermore, surface modification with bioactive molecule coatings is one of the strategic approaches used to enhance osseointegration. Among the candidates for bioactive molecules, secretory leukocyte protease inhibitor (SLPI) has emerged as a promising coating agent. Previous studies revealed that SLPI promotes osteoblast cell adhesion, proliferation, and differentiation [5,6].

SLPI is a multifunctional non-glycosylated protein that is widely distributed in mucous secretion [7]. While its primary function is as a serine protease inhibitor, it also possesses anti-inflammatory properties by inhibiting the nuclear factor-kappa B (NF-κB) signaling pathway [8]. Moreover, SLPI exhibits potent broad-spectrum antimicrobial activity, including antibacterial, by destabilizing the bacterial cell wall due to its strong positive charge [7]. Beyond its role in immune regulation and antimicrobial activities, SLPI has remarkable potential for osteogenic activity. Previous studies revealed that SLPI enhances osteoblast cell adhesion through the focal adhesion kinase (FAK) signaling pathway and upregulates the expression of adhesion molecules, including integrin subunits [5,6]. Moreover, SLPI significantly upregulated the expression of osteogenic differentiation genes and enhanced mineralization [5,9]. Although previous in vitro studies have demonstrated the osteogenic potential of SLPI-coated titanium, the in vivo osseointegration efficacy of this surface modification has not yet been investigated. Therefore, this is the first study to evaluate the biological performance of SLPI-coated titanium implants in an in vivo model.

The aim of the present study was to evaluate the osseointegration efficacy of SLPI-coated titanium screws in a Sprague–Dawley rat model. The assessment was performed using micro-computed tomography (micro-CT), histomorphometric analysis, and removal torque testing.

## 2. Materials and Methods

### 2.1. Chemicals and Reagents

Recombinant human secretory leukocyte protease inhibitor (rhSLPI) was purchased from Sino Biology Inc. (Paoli, PA, USA). Other pharmaceutical agents utilized included Isoflurane, Mepivacaine with 1:100,000 epinephrine, morphine, meloxicam, and enrofloxacin.

### 2.2. The Sample Preparation

**Figure 1:**
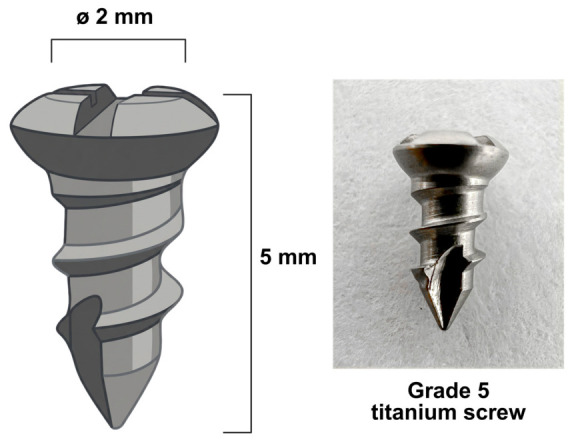
The illustration of a grade 5 titanium screw. The size of the screw is 2 mm in diameter (∅) and 5 mm in total length.

The commercial smooth-surface grade 5 titanium screws (Ti), with a standard machined procedure and without any prior bio-functional coatings or specialized roughening treatments, used to implant in the tibia bone, were purchased from MEDI US Limited Company, Bangkok, Thailand. The external diameter of the screw body is 2 mm, and the total length is 5 mm (Figure 1). The Ti were sterilized by autoclave and dried in a hot air oven overnight. Then, the Ti was fully immersed in rhSLPI solution at a concentration of 10 µg/mL rhSLPI in ultrapure water at 4 °C for 24 h, which, based on the previous in vitro study, allowed physical adsorption of the protein onto the titanium surface [5]. After the preparation process, the implants were categorized into two experimental groups: the uncoated titanium screw (Ti), which served as the control group, and the rhSLPI-coated titanium screw (TiS).

### 2.3. Sample Morphology

The surface morphology of the Ti screw was observed by a field emission scanning electron microscope (FE-SEM, JSM-IT800; JEOL, Tokyo, Japan). Ti screws were placed on the stub and observed under the FE-SEM at 30× and 500× magnification for the surface morphology.

### 2.4. Determination of rhSLPI on Ti Surface by ELISA

The presence of rhSLPI on the Ti surface was determined by using a sandwich enzyme-linked immunosorbent assay (ELISA, Abcam, Cambridge, UK). After coating with rhSLPI, the Ti screws were rinsed three times with PBS and then placed into the microcentrifuge tube. The antibody mixture was added to the samples and incubated at 25 °C for 1 h. Subsequently, the samples were subjected to three washes with wash buffer, followed by the application of the TMB development solution. Following a ten-minute incubation period, the stop solution was added. Absorbance was measured at 450 nm by using a microplate spectrophotometer.

### 2.5. Animals and Animal In Vivo Protocol

A total of 36 male, ten-week-old Sprague–Dawley (SD) rats were obtained from M-CLEA Nomura Siam (Bangkok, Thailand). The animals were housed at the Laboratory Animal Center, Chiang Mai University, under the following optimal conditions: temperature of 21 ± 1 °C, dark/light cycle for 12 h, and 50% humidity. The animal experiments in this study were conducted according to the Guidance on the Operation of the Animals (Scientific Procedures) Act 1986 and the World Health Organization Guidelines for Breeding and Care of Laboratory Animals. All the protocols were approved by the committee of the Laboratory Animal Center, Office of Research Administration, Chiang Mai University (Chiang Mai, Thailand), Protocol No. 2567/RT-0018.

**Figure 2:**
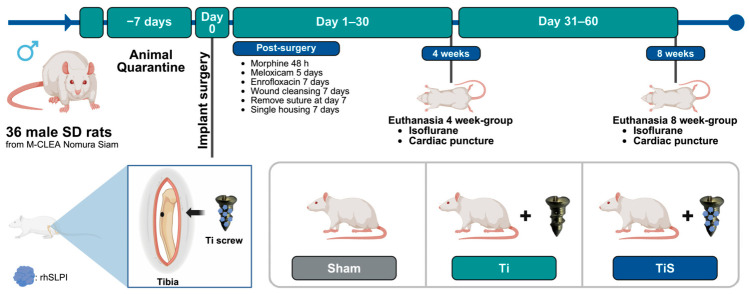
The experimental timeline and animal grouping. The animals were implanted for 4 weeks and 8 weeks post-surgery. The animals were randomly divided into 3 groups, including sham, uncoated Ti (Ti), and rhSLPI-coated Ti (TiS).

The animals were randomly allocated into 3 groups of 12 rats per group as follows: sham, uncoated Ti screws (Ti), and rhSLPI-coated Ti screws (TiS). In each group of the study, the animals were divided into 2 time-point studies, 4 weeks and 8 weeks. After the implantation and healing period, the animals were euthanized, and the tibia bones were collected to assess the osseointegration (Figure 2).

### 2.6. Implant Surgery and Post-Surgery

**Figure 3:**
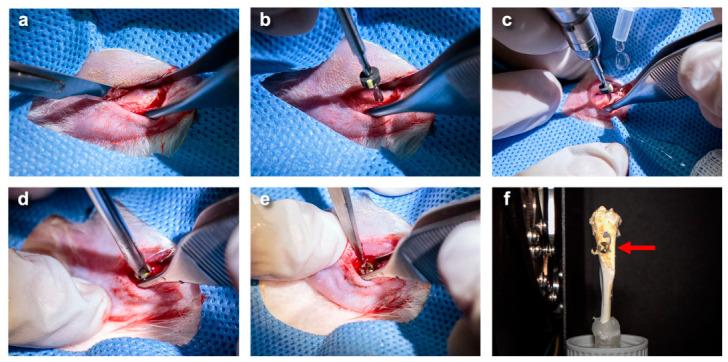
The demonstration of the surgical procedure. (**a**–**e**) The animal preparation and surgical steps were performed under standard aseptic technique and in accordance with ethical guidelines. (**f**) The titanium screw was implanted into the tibial metaphysis (red arrow).

The surgical procedure followed the Laboratory Animal Center-approved protocol and was conducted by BB, a licensed veterinary surgeon (Figure 3). Initially, anesthesia was performed by inducing 4% isoflurane (99% purity, Baxter Healthcare Corporation, Deerfield, IL, USA) with 96% oxygen for induction and a maintenance concentration of 2% during surgery. Then, the left hind limb of the SD rat was shaved and cleaned with 70% ethanol and 10% povidone iodine. Subsequently, a subcutaneous injection with 2% mepivacaine with 1:100,000 epinephrine (Septodont, Saint-Maur-des-Fossés, France) was administered at a dose of 7 mg/kg. Next, the hind limb was incised on the medial aspect of the hind limb knee to expose the tibial metaphysis (Figure 3a). A hole with a diameter of 2 mm was created using a dental drill (Figure 3b). Subsequently, a screw was implanted into the hole, except in the sham group, which underwent the same surgical procedure without implantation (Figure 3c–e). After implantation, the soft tissue was closed with absorbable sutures, and the skin was sutured using 4–0 nylon. The implants remained in the animals for 4 and 8 weeks after surgery. Post-surgery, the animals obtained 10 mg/mL of morphine (M&H manufacturing, Samut Prakan, Thailand) at a dose of 2.5 mg/kg every 12 h for 48 h and 5 mg/mL of meloxicam (Metacam^®^, Boehringer Ingelheim Animal Health, Ingelheim am Rhein, Germany) at a dose of 2 mL/kg after surgery and 1 mL/kg for 5 days. Moreover, the 50 mg/mL of enrofloxacin (Baytril^®^, Bayer A/S, Animal Health Division, Copenhagen, Denmark) was subcutaneously administered at a dose of 10 mg/kg every 24 h for 7 days to prevent the bacterial infection. Post-operation, the surgical site was disinfected with povidone iodine daily for 7 days, and an Elizabethan collar (E-collar) was applied to prevent self-mutilation of the wound. The suture was removed on day 7 post-surgery, following confirmation of wound closure and absence of infection.

### 2.7. Sample Harvesting

At 4 and 8 weeks post-implantation, the animals were euthanized by deep anesthesia with 4% isoflurane followed by cardiac puncture. The blood samples were collected to evaluate the complete blood count (CBC) and serum biochemical biomarkers, including aspartate aminotransferase (AST) for liver function, blood urea nitrogen (BUN) and creatinine for kidney function, and troponin I as a cardiac biomarker, by an automate analyzer. Subsequently, the left tibiae were harvested and randomly preserved into two groups. The first group was preserved in neutral buffered formalin (4% formaldehyde in a phosphate-buffered solution, pH 7.0) for micro-computed tomography (micro-CT) and histological evaluation for osteointegration, and the second group was preserved as a fresh-frozen sample for the removal torque test.

### 2.8. Micro-CT Analysis for Bone Volume Fraction (BV/TV)

After 2 weeks of fixation, micro-CT was performed by using a micro-CT scanner (NEOSCAN N80, Mechelen, Belgium) with a pixel resolution of 5 µm. The tibia was vertically placed in the sample holder, and the 0.5 mm copper filter was applied for optimal contrast. The scanning parameters were adjusted to 106 kV for source voltage and 120 µA for source current. Furthermore, image reconstruction was performed using Neoscan software (https://neoscan.com/software/neoscan-software/, accessed on 17 April 2026). The quantitative data of BV/TV were analyzed using Dragonfly 3D world software (version 2025, Comet Technologies Canada Inc., Montréal, QC, Canada). The region of interest (ROI) was defined as a concentric cylindrical volume of bone surrounding the implant. The ROI was segmented and evaluated at a distance of 60 to 250 µm from the implant surface to eliminate metallic artifacts from the titanium surface [2].

### 2.9. The Histomorphology Evaluation

Following micro-CT scanning, histomorphology was performed to confirm the bone regeneration around the implant according to micro-CT findings. Firstly, the samples were decalcified by using a slow decalcification method with an ethylenediaminetetraacetic acid (EDTA) solution [10]. Specimens were immersed in 10% EDTA solution under agitation at 25 °C until the bone tissue became soft and pliable. The Ti screws were carefully removed, and the specimens were rinsed with PBS for 30 min. The samples were dehydrated through a graded ethanol series and embedded in paraffin. The specimens were sectioned into 7 µm slices using a microtome. Finally, the histological sections were stained with hematoxylin and eosin (H&E).

### 2.10. The Removal Torque Test

**Figure 4:**
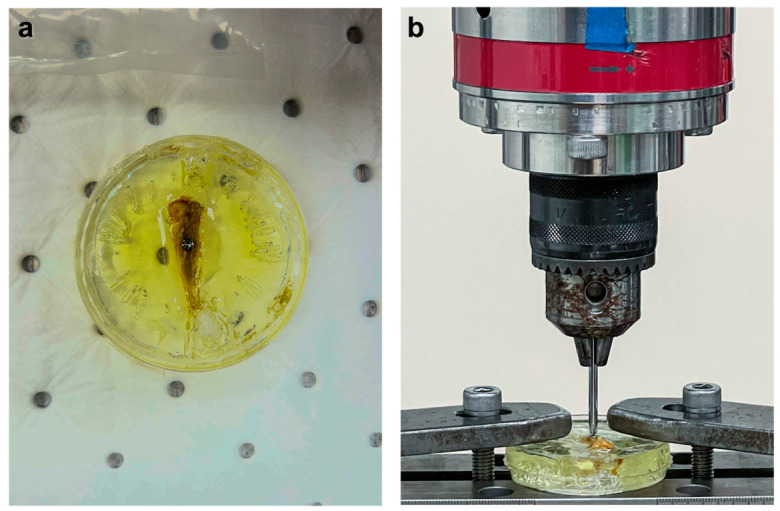
Removal torque test procedure. (**a**) The sample was embedded in a resin block to ensure stability during testing. (**b**) The torque machine applied counterclockwise rotation to detach the Ti screw from the bone.

After euthanasia, the fresh-frozen specimens were assessed to evaluate the removal torque test (Figure 4). Initially, the overlying soft tissue and bone were removed to expose the implant. The samples were then embedded in resin to stabilize the position. The removal torque was performed using an Electropuls^®^ E10000 torque machine (Instron, Norwood, MA, USA). A counterclockwise rotation was applied to the implant until the bone–implant interface completely detached. The maximum torque value was recorded in Newton centimeters (N.cm) [11].

### 2.11. Statistical Analysis

Statistical analysis was performed using commercially available software (GraphPad Prism version 10, San Diego, CA, USA). All data are expressed as the mean ± SD. All comparisons were assessed for significance using an unpaired *t*-test or ANOVA, followed by, when appropriate, the Tukey–Kramer test. A *p*-value of less than 0.05 was considered significant.

## 3. Results

### 3.1. Titanium Screw Characterization

**Figure 5:**
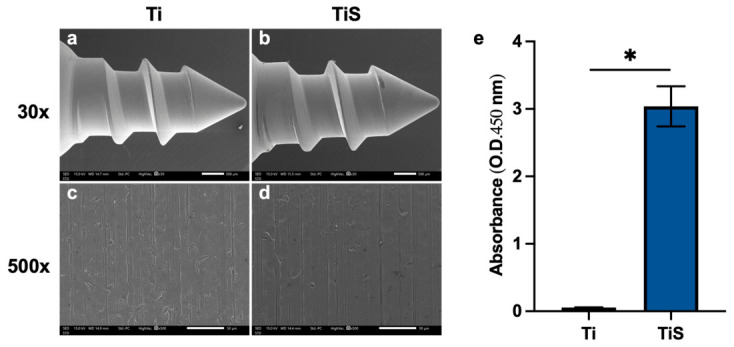
The characterization of the titanium screw. (**a**–**d**) The morphology of Ti screws was observed under FE-SEM. (**e**) The confirmation of rhSLPI coating was evaluated by ELISA. * *p* < 0.05 vs. control (unpaired *t*-test) (*n* = 4 per group).

The field emission scanning electron microscope (FE-SEM) was used to observe surface morphology. The results demonstrated that both Ti and TiS exhibited a smooth surface with minor scratch lines (Figure 5a–d). The ELISA confirmed the successful immobilization of the protein, revealing a significantly high amount of rhSLPI on the TiS surface (3.03 ± 0.297). In contrast, the uncoated Ti (Ti) exhibited minimum background absorbance inherent to the assay (0.05 ± 0.003), confirming the absence of the specific protein on the control implants (Figure 5e).

### 3.2. Evaluation of Systemic Toxicity

To evaluate the systemic toxicity of the rhSLPI coating in an in vivo model, the hematological and serum biochemical parameters were analyzed at 4 and 8 weeks post-implantation. Hematological parameters, including the quality and quantity of red blood cell (RBC), hemoglobin (HGB), hematocrit (HCT), white blood cell (WBC) counts, and platelet counts, are demonstrated in Table 1. At both 4 and 8 weeks, all hematological values in the TiS group were comparable to those of the Ti and the sham groups, which showed no statistically significant differences.

**Figure 6:**
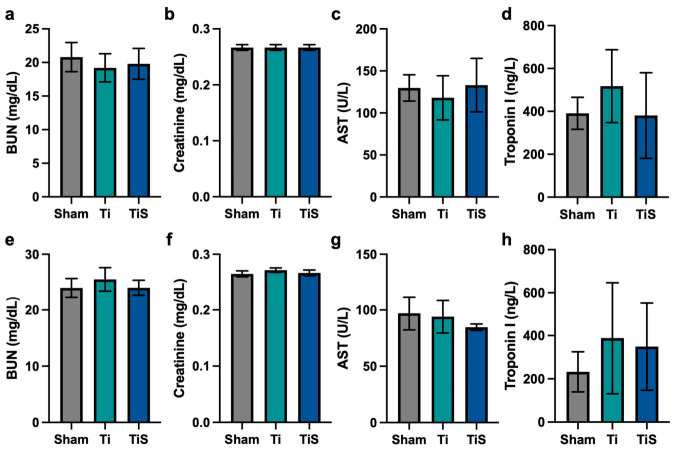
Post-implant blood parameter. (**a**–**d**) The biochemical parameter for 4 weeks and (**e**–**h**) for 8 weeks.

Moreover, serum biochemical analysis was performed to assess the toxicity on major organs, including renal function, hepatic function, and cardiac function (Figure 6). Firstly, the renal function, including blood urea nitrogen (BUN) (Figure 6a,e) and creatinine (Figure 6b,f) of TiS, exhibited no significant differences compared to the Sham and Ti groups at both time points. Furthermore, aspartate aminotransferase (AST) was evaluated for liver function tests. The result showed that there are no significant differences across all groups at both time points (Figure 6c,g). Next, the cardiac function, troponin I level, exhibited no significant elevation in TiS compared to Ti and Sham at 4 or 8 weeks (Figure 6d,h).

### 3.3. Enhancement of Osseointegration

**Figure 7:**
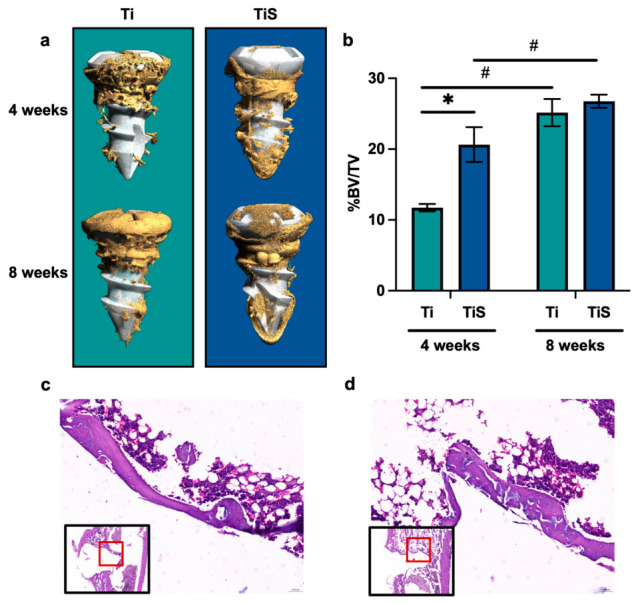
The bone formation on the titanium screw. (**a**) The representative 3D micro-CT reconstruction showing bone (yellow) around the Ti implant (white) at 4 weeks and 8 weeks post-implantation. (**b**) The percentage of bone fraction (%BV/TV) was analyzed in Dragonfly 3D World software. (**c**) Representative histomorphological image of new bone formation around the Ti implant surface at 4 weeks post-surgery. (**d**) Representative histomorphological image of new bone formation around the TiS implant surface at 4 weeks post-surgery. For both (**c**) and (**d**), the lower-left insets display the macroscopic overview of the section (black box) and indicate the specific region of interest (red box) magnified in the main panel. * *p* < 0.05 vs. control, and # *p* < 0.05 for the comparison between 4 and 8 weeks post-surgery within the same group (Unpaired *t*-test) (*n* = 3 per group).

Micro-computed tomography (micro-CT) was performed to reveal the amount of bone around the implant at 4 and 8 weeks post-implantation. The qualitative 3D reconstruction images revealed that the TiS exhibited more extensive bone formation surrounding the implant threads compared to Ti (Figure 7a). The quantitative analysis of the percentage of bone fraction (%BV/TV) was analyzed by using Dragonfly 3D World (Figure 7b). At 4 weeks post-implantation, the results demonstrated that TiS (20.64% ± 2.452%) showed a significantly higher %BV/TV compared to Ti, the control group (11.73% ± 0.524%). At 8 weeks, both groups demonstrated a significant increase in %BV/TV compared to 4 weeks post-implantation. However, the %BV/TV between TiS and Ti at 8 weeks exhibited no significant difference. Moreover, histomorphology observation at 4 weeks post-implantation was performed to qualitatively confirm the micro-CT findings, verifying the presence of newly formed bone tissue within the peri-implant region in both groups (Figure 7c,d).

### 3.4. Biomechanical Stability

**Figure 8:**
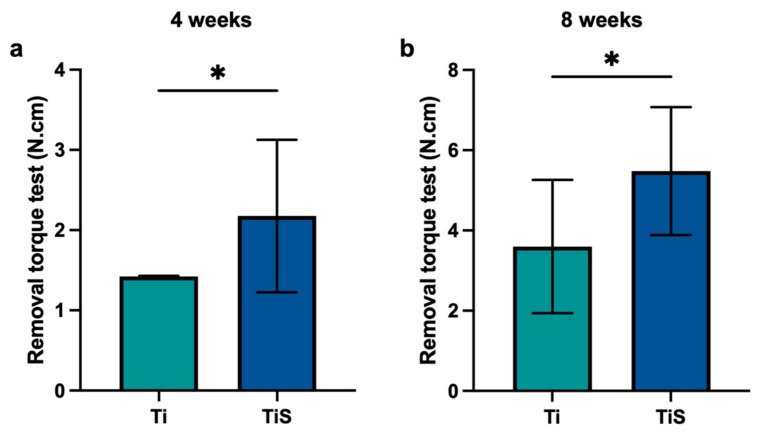
The removal torque of the implant. The torque test was performed by using a torque machine at (**a**) 4 weeks and (**b**) 8 weeks post-implant. The quantitative data were represented in Newton centimeters (N.cm). * *p* < 0.05 vs. control (Unpaired *t*-test) (*n* = 3 per group).

The biomechanical stability of the implants was assessed using a removal torque test at 4 and 8 weeks (Figure 8). At 4 weeks post-implantation, the TiS (2.17 ± 0.951 N.cm) demonstrated a significantly higher removal torque value compared to the control group (1.42 ± 0.008 N.cm) (Figure 8a). Moreover, the TiS (5.48 ± 1.593 N.cm) exhibited a significantly higher removal torque compared to the Ti (3.59 ± 1.661 N.cm) at 8 weeks (Figure 8b).

## 4. Discussion

The grade 5 titanium screws (Ti) were coated with 10 µg/mL of recombinant human secretory leukocyte protease inhibitor (rhSLPI). Then, the surface morphology was characterized under FE-SEM, and the presence of protein was evaluated by ELISA to confirm successful coating. The FE-SEM results revealed the scratch lines on the screw surface, attributed to the machining process in both Ti and TiS groups. In this study, a simple coating technique was employed to physically adsorb SLPI onto the screw surface through non-covalent interactions. Moreover, the presence of rhSLPI protein on TiS was detected, confirming the successful coating technique, which is consistent with the previous study [5].

Although the current study primarily established local osseointegration efficacy, hematological and biochemical analyses were performed to ensure systemic inflammation, infection, or hematotoxicity [12]. The CBC demonstrated that the total white blood cell (WBC) count and other blood indices in the TiS group were not different from the Sham and Ti groups at both 4 and 8 weeks post-implantation. The finding suggests that the SLPI coating on titanium did not trigger an immune response or chronic inflammation, which is consistent with the immune-suppression and anti-inflammation effects of SLPI [7]. Moreover, the biochemical parameters were used to evaluate the primary organ toxicity. The liver and kidneys are the major organs that respond to the metabolism, detoxification, and excretion of biochemical waste [13]. The absence of significant differences in BUN and creatinine levels indicates normal renal function [14]. Moreover, the normal level of AST in TiS, which was comparable to that of the Sham and Ti groups, indicated no toxicity to hepatic function [15]. Additionally, the absence of elevation in troponin I, which is a highly specific and sensitive cardiac biomarker, indicated that the SLPI coating showed no cardiotoxic effect [16]. Furthermore, since AST is not exclusively distributed in the liver but is also present in the kidneys and heart, the normal AST levels observed in this study corroborate the renal and cardiac biomarker findings [17]. This collectively suggests that the SLPI coating induces no acute systemic hematological and biochemical alterations within the 8-week evaluation period. The findings revealed that the physical adsorption of rhSLPI on titanium causes no observed acute systemic toxicity in the short term. Thus, SLPI could be a candidate for future dental and orthopedic applications.

Micro-CT was performed to reveal and evaluate the osteogenic efficacy of SLPI coating. The histomorphology was observed to confirm the new bone formation on the Ti screw surface. One finding was the significantly higher %BV/TV in the TiS group at 4 weeks, which indicates that rhSLPI accelerates early-stage osseointegration. This rapid bone formation is clinically crucial for implant stability and reducing the healing period [3]. Osseointegration is a complex biological cascade comprising four sequential stages: protein adsorption, inflammatory response, osteogenic cell adhesion, and bone formation [18]. As protein adsorption is the initial stage for cellular–implant surface interaction, a previous study revealed that SLPI-coated titanium disks demonstrated significantly enhanced surface hydrophilicity [5]. Additionally, a previous in vivo study suggested that SLPI exhibited potent anti-inflammation that suppressed the excessive immune response, which might allow an earlier shift toward the osteogenic stage [7]. In the cell adhesion stage, the evidence demonstrated the positive effect of SLPI in an in vitro osteoblast cell adhesion that could be one of the factors to enhance osseointegration [6,19]. Moreover, current findings exhibited that SLPI-coated titanium promoted higher bone formation compared to conventional titanium, which is consistent with the previous in vitro and in vivo studies that SLPI-coated titanium disks enhance differentiation and bone healing [5,20]. Collectively, previous studies and current findings of SLPI coating on titanium effectively enhance osseointegration in the early stages of healing. Therefore, SLPI could emerge as a promising coating agent for medical implants to improve clinical success.

The removal torque test serves as an evaluation of osseointegration [21]. In this study, the significantly higher removal torque values observed in the TiS at both 4 and 8 weeks exhibited effectively enhanced osseointegration [22]. The significantly higher torque removal value in TiS at 4 weeks is consistent with the micro-CT finding of increased new bone formation around the implant, resulting in increased mechanical stability in the early stage [11]. At 8 weeks post-implantation, the findings exhibited higher biomechanical stability of the TiS, whereas the quantity of bone in the micro-CT revealed no significant differences between groups. This discrepancy suggested that the SLPI coating promotes higher bone maturation of new bone, resulting in a higher torque value even when the total volume of bone is comparable to that of the control group. The finding revealed that the SLPI coating could enhance not only early-stage osseointegration but also the stability of the implant.

The present study investigated the efficacy of SLPI coating on titanium in an in vivo model, demonstrating its potential as a promising coating agent for dental and orthopedic implants. Nevertheless, there are some issues that are considered limitations of the current study. The findings revealed the potential of rhSLPI coating to enhance early osseointegration and biomechanical stability. However, simple physical adsorption might not be sufficient for real clinical settings, necessitating advanced coating techniques for long-term implantation. This binding mechanism is hypothesized to be primarily driven by electrostatic interactions. Since rhSLPI is a highly cationic protein with an alkaline isoelectric point (pI > 9), it possesses a net positive charge at a neutral pH [7]. Consequently, SLPI could adsorb onto the naturally occurring, negatively charged titanium dioxide (TiO_2_) passivation layer through electrostatic attraction, alongside potential van der Waals forces and hydrogen bonding [23]. Stability, release kinetics of protein coating under physicochemical conditions, shelf-life, gradual desorption profiles in physiological fluids, and the potential loss of the coating due to mechanical shear stress during surgical insertion into the tibia were not evaluated. Furthermore, the current functionalization relied on physical adsorption, and comprehensive surface characterization, including wettability, surface roughness, and in vitro coating stability profiles, was not evaluated in the present study. Moreover, this study used healthy adult rats for generating the study model, which does not reflect the complex pathophysiological conditions often encountered in clinical scenarios, such as aging, hyperglycemia, or osteoporosis [24,25], which need to be considered for future applications in real clinical settings. Additionally, the potential influence of hormonal fluctuations in females was not evaluated despite its known impact on bone metabolism and implant integration [26]. Additionally, the current study provides only short-term hematological and biochemical data. Therefore, comprehensive evaluations of long-term safety, potential immunogenicity, and detailed local tissue responses at the bone–implant interface are necessary. Furthermore, the evaluation of systemic inflammation was based on WBC count, without the quantitative measurement of specific systemic inflammatory cytokines such as tumor necrotic factor-α (TBF-α), interleukin 1β (IL-1β), or IL-6 [27]. Moreover, the limitations of this study are peri-inflammation and molecular evaluation. Additionally, the current study was focused on osseointegration and toxicity, whereas the SLPI provides anti-inflammatory or antimicrobial activities [7]. Although these parameters were not assessed in vivo, the TiS implant nevertheless demonstrated substantial potential for enhancing osseointegration. In addition, due to the specific anatomical location of the implantation in this study, the evaluation of trabecular microarchitectural parameters such as trabecular number (Tb.N), trabecular thickness (Tb.Th), and trabecular space (Tb.Sp) via micro-CT was limited. Although we were restricted to evaluating the bone volume fraction (%BV/TV), the significantly higher removal torque values observed in the TiS group strongly suggest a functional enhancement in the quality and strength of the bone–implant interface. For future assessments targeting comprehensive trabecular bone quality, implants should be inserted at a more proximal location where cancellous bone is abundant. Additionally, the histological protocol involving decalcification necessitated the physical removal of the implant prior to embedding. This process disrupts the true bone–implant interface. Therefore, the histological data serve primarily to qualitatively verify the presence of adjacent peri-implant bone tissue rather than providing bone–implant contact (BIC).

## 5. Conclusions

This current study is the first in vivo proof-of-concept study on the evaluation of human recombinant secretory leukocyte protease inhibitor (rhSLPI) coating on titanium via simple physical adsorption for enhancing osteointegration. The findings demonstrated that the SLPI-coated titanium screw (TiS) exhibited significantly higher peri-implant bone volume fractions (%BV/TV) and greater removal torque compared to the uncoated titanium screw (TiS), which indicated that rhSLPI enhances early osseointegration and biomechanical stability. Furthermore, the 8-week implantation demonstrated no acute hematological and biochemical alterations, suggesting a favorable safety profile.

## Figures and Tables

**Table 1:** The complete blood count (CBC) parameters.

Groups	Time (Weeks)	RBC (×10^6^/µL)	HGB (g/dL)	HCT (%)	MCV (fL)	MCH (pg)	MCHC (g/dL)	RDW (%)	Platelet Count (×10^5^ cell/mm^3^)	WBC (×10^3^ cell/mm^3^)
Sham	4	7.72 ± 0.502	15.95 ± 1.046	42.00 ± 3.464	54.35 ± 1.851	20.66 ± 0.413	38.05 ± 1.122	14.46 ± 0.332	5.41 ± 1.985	7.41 ± 1.088
	8	8.14 ± 0.271	16.00 ± 0.953	43.11 ± 2.807	52.83 ± 1.807	19.61 ± 0.584	37.13 ± 1.139	14.83 ± 0.454	7.64 ± 0.596	6.72 ± 0.780
Ti	4	6.25 ± 1.981	13.33 ± 4.414	34.31 ± 11.172	54.76 ± 1.179	21.25 ± 0.763	38.76 ± 1.123	14.13 ± 0.338	4.37 ± 3.049	5.89 ± 2.610
	8	8.17 ± 0.372	15.70 ± 0.657	41.58 ± 1.581	50.90 ± 1.385	19.21 ± 0.611	37.76 ± 0.750	15.46 ± 0.720	7.66 ± 0.826	6.24 ± 1.019
TiS	4	7.68 ± 0.459	15.88 ± 0.458	40.98 ± 1.635	53.40 ± 1.808	20.73 ± 0.691	38.81 ± 1.010	14.16 ± 0.355	4.84 ± 2.627	6.97 ± 0.915
	8	7.74 ± 0.275	15.91 ± 0.508	41.56 ± 0.484	53.73 ± 1.537	20.58 ± 0.923	38.28 ± 1.290	14.88 ± 0.263	7.89 ± 0.616	6.42 ± 1.058

## Data Availability

The raw data supporting the conclusions of this article will be made available by the authors on request.

## Abbreviations

The following abbreviations are used in this manuscript:

AST	Aspartate aminotransferase
BUN	Blood urea nitrogen
BV/TV	Bone volume fraction
CBC	Complete blood count
EDTA	Ethylenediaminetetraacetic acid
ELISA	Enzyme-linked immunosorbent assay
FAK	Focal adhesion kinase
FE-SEM	Field emission scanning electron microscope
H&E	Hematoxylin and Eosin
HCT	Hematocrit
HGB	Hemoglobin
IL-1β	Interleukin 1 β
IL-6	Interleukin 6
MCH	Mean corpuscular hemoglobin
MCHC	Mean corpuscular hemoglobin concentration
MCV	Mean corpuscular volume
micro-CT	Micro-computed tomography
N.cm	Newton-centimeters
NF-κB	Nuclear factor-kappa B
PBS	Phosphate-buffered solution
RBC	Red blood cell
RDW	Red cell distribution width
rhSLPI	Recombinant human secretory leukocyte protease inhibitor
ROI	Region of interest
SD Rats	Sprague–Dawley rat
SLPI	Secretory leukocyte protease inhibitor
TBF-α	Tumour necrotic factor-α
Ti	Uncoated Ti screw
TiS	rhSLPI-coated Ti screw
TMB	TMB development solution
WBC	White blood cell
